# Critical Roles of Spätzle5 in Antimicrobial Peptide Production Against *Escherichia coli* in *Tenebrio molitor* Malpighian Tubules

**DOI:** 10.3389/fimmu.2021.760475

**Published:** 2021-12-16

**Authors:** Maryam Ali Mohammadie Kojour, Tariku Tesfaye Edosa, Ho Am Jang, Maryam Keshavarz, Yong Hun Jo, Yeon Soo Han

**Affiliations:** ^1^ Department of Applied Biology, Institute of Environmentally-Friendly Agriculture (IEFA), College of Agriculture and Life Sciences, Chonnam National University, Gwangju, South Korea; ^2^ Ethiopian Institute of Agricultural Research, Ambo Agricultural Research Center, Ambo, Ethiopia; ^3^ Department of Evolutionary Biology, Institute of Biology, Free University of Berlin, Berlin, Germany

**Keywords:** *Tenebrio molitor*, innate immune response, spätzle 5, antimicrobial peptides, NF-κB, Toll signaling pathway, Malpighian tubules

## Abstract

The dimeric cytokine ligand Spätzle (Spz) is responsible for Toll pathway activation and antimicrobial peptide (AMP) production upon pathogen challenge in *Tenebrio molitor*. Here, we indicated that *Tm*Spz5 has a functional role in response to bacterial infections. We showed that the highest expression of *TmSpz5* is induced by *Candida albicans*. However, *TmSpz5* knockdown reduced larval survival against *Escherichia coli* and *Staphylococcus aureus*. To evaluate the molecular mechanism underlying the observed survival differences, the role of *TmSpz5* in AMP production was examined by RNA interference and microbial injection. *T. molitor* AMPs that are active against Gram-negative and -positive bacteria, including *Tm*tenecins, *Tm*attacins, *Tm*coleoptericins, *Tm*taumatin-like-proteins, and *Tm*cecropin-2, were significantly downregulated by *TmSpz-5* RNAi in the Malpighian tubules (MTs) following a challenge with *E. coli* and *S. aureus*. However, upon infection with *C. albicans* the mRNA levels of most AMPs in the ds*TmSpz5*-injected group were similar to those in the control groups. Likewise, the expression of the transcription factors NF-κB, *TmDorX2*, and *TmRelish* were noticeably suppressed in the MTs of *TmSpz5*-silenced larvae. Moreover, *E. coli*-infected *TmSpz5* knockdown larvae showed decreased antimicrobial activity in the MTs and hindgut compared with the control group. These results demonstrate that *Tm*Spz5 has a defined role in *T. molitor* innate immunity by regulating AMP expression in MTs in response to *E. coli*.

## Introduction

Insects have been the largest and most diverse class over millions of years of evolution and have adapted to survive in a vast range of ecological territories (1–3). Owing to their exposure to various pathogen sources including bacteria, fungi, parasites, and viruses, they have evolved several multifunctional defense mechanisms, making them an exceptional model for immunity studies (4, 5). Unlike mammals, insects do not have an adaptive immunity (2). However, they do possess a functional innate immune system, involving both humoral and cellular immune responses (6). Cellular immunity, mediated by hemocytes (insect blood cells), involves nodulation (7), encapsulation (8), and phagocytosis (9). Humoral immune response, on the other hand, is mainly mediated by fat bodies (the equivalent of the mammalian liver) and soluble plasma proteins. The production of antimicrobial peptides (AMPs) is the main determinant of humoral immunity (6). Following invader recognition, AMP production is stimulated by the activation of two major signaling pathways, the immune deficiency (Imd) and Toll pathways (6, 10).

The Toll signaling pathway was initially identified as a dorso-ventral axis establishment regulator during embryonic development in *Drosophila melanogaster* (11). Since then, extensive molecular and mapping studies have provided insight into the roles of the Toll pathway and its components in the *Drosophila* immune system. The key activator of the Toll transmembrane-associated receptor is the endogenous cytokine-like polypeptide Spätzle (Spz) (12). Invader detection by peptidoglycan recognition proteins (PGRPs) or β-1,3-glucan recognition protein (βGRP)/Gram-negative-binding proteins (GNBPs) in *Drosophila* leads to a proteolytic cascade that eventually results in Spätzle cleavage and activation. Spätzle can then bind to the Toll receptor and activate downstream signaling pathways, leading to AMP production (10, 13, 14). In addition to its role in *D. melanogaster*, Spätzle has been shown to have significant roles in different species and taxa, including mosquitoes (15–17), *Manduca sexta* (18), *Bombyx mori* (19), shrimp (20), and *Tenebrio molitor* (21).

Comprehensive biochemical studies of innate immunity claim for a relatively large insect model to enable the collection of sufficient hemolymph samples. Thus, in the last two decades, *T. molitor* has become a common model for biochemical and molecular studies on innate immunity pathways and their components (22).

Toll signaling in *T. molitor* is activated when PGRP-SA and GNBP1 recognize *meso*-diaminopimelic acid (DAP)-type peptidoglycan (PGN) of Gram-negative bacteria and some *Bacillus* species, and the lysine-type peptidoglycan of Gram-positive bacteria (21, 23–26). However, in *Drosophila*, the PGRP-SA/GNBP1 complex solely recognizes Gram-positive bacterial and fungal infections, whereas Gram-negative bacteria can be sensed by the alternative receptors PGRP-LC and PGRP-LE and triggers the Imd signaling pathway (6, 27–31). Following recognition, a proteolytic cascade activation, including modular serine protease (MSP), Spz-processing enzyme (SPE)-activating enzyme (SAE), and SPE, leads to the cleavage of Spätzle zymogen, and eventually, mature Spätzle recruitments to the Toll receptor (32, 33). Upon Spz–Toll association in *T. molitor*, an intracellular cascade is activated, resulting in the engagement of myeloid differentiation factor 88 (MyD88), tube, pelle, pellino, and tumor necrosis factor receptor associated factor (TRAF). This ultimately leads to the binding of cactin to cactus, a dorsal-related immunity factor (Dif) and dorsal inhibitor (34, 35). These transcription factors translocate to the nucleus (36, 37), where they bind to NF-κB-response elements and induce AMP genes transcription (38–43). We have identified nine *Spätzle* genes (*TmSpz-like*, *-1b*, *-3*, *-4*, *-5*, *-6*, *-7*, *-7a*, and -*7b*) in *T. molitor.* However, the functional importance of these isoforms is poorly understood. To date, only two *T. molitor Spätzle* genes (*TmSpz4* and *TmSpz6*) have been functionally characterized (44, 45). In this study, we focused on the immunological significance of *Tm*Spz5 against microbial infection (**Supplementary Table 1**).

## Materials and Methods

### Insect Rearing

*T. molitor* larvae were reared under dark conditions at 26 ± 1°C and 60 ± 5% relative humidity in an environmental chamber established in the laboratory. Larvae were fed an artificial diet consisting of 1.1 g sorbic acid, 1.1 ml propionic acid, 20 g bean powder, 10 g brewer’s yeast powder, and 200 g wheat bran in 4,400 ml distilled water. The feed was autoclaved at 121°C for 15 min and fed to healthy 10th–12th instar larvae for all experiments.

### Microorganism Preparation

The Gram-negative bacterium *Escherichia coli* (strain K12), Gram-positive bacterium *Staphylococcus aureus* (strain RN4220), and fungus *Candida albicans* (strain AUMC 13529) were used as pathogenic invaders. *E. coli* and *S. aureus* were cultured in Luria–Bertani (LB) broth, and *C. albicans* was cultured in Sabouraud’s dextrose broth overnight at 37°C. The microorganisms were harvested and washed twice in phosphate-buffered saline (PBS; pH 7.0) and then centrifuged at 3,500 × g for 15 min. Subsequently, the samples were suspended in PBS, and concentrations were measured at 600 nm (OD_600_) by spectrophotometry (Eppendorf, Hamburg, Germany). *E. coli* and *S. aureus* were diluted to 1 × 10^6^ cells/µl, and *C. albicans* was diluted to 5 × 10^4^ cells/µl for immune challenge studies.

### Computational Sequence Analysis of *TmSpz5*

The *TmSpz5* gene sequence (accession number: MW916536) was obtained from the *T. molitor* RNAseq analysis (unpublished) and NCBI Expressed Sequence Tag (EST) database. The *Tribolium castaneum* Spz5 amino acid sequence (accession number: XP_008193940.1) was used as the query for identification by local-tblastn searches. The full-length open reading frame (ORF) and deduced amino acid sequences of *TmSpz5* were analyzed using BLASTp (NCBI; https://blast.ncbi.nlm.nih.gov/Blast.cgi). The domain architectures of the protein sequences were retrieved using InterProScan (https://www.ebi.ac.uk/interpro/search/sequence-search). Signal peptides were predicted using the SignalP 5.0 server (http://www.cbs.dtu.dk/services/SignalP/).

A multiple-sequence alignment of the *Tm*Spz5 amino acid sequence with representative Spätzle amino acid sequences from other insects (retrieved from GenBank) was generated using ClustalX 2.1 (46). Estimation of the percent identity and phylogenetic analyses were performed using ClustalX 2.1 (pim as the output file) and MEGA version 7.0 (47), respectively. Evolutionary relationships were inferred using the neighbor-joining method (48), and the bootstrap consensus tree was inferred from 1,000 replicates. Several protein sequences were used to generate the phylogenetic tree, including those of *Tc*Spz5, *Tribolium castaneum* spätzle 5 isoform X1 (XP_008193940.1); *Tc*Spz5, *Tribolium castaneum* spätzle 5 isoform X2 (XP_015836109.1); *At*Spz5like, *Aethina tumida* spätzle 5-like (XP_019879590.1); *So*Spz5like, *Sitophilus oryzae* spätzle 5-like (XP_030767938.1); *Ms*Spz5, *M. sexta* spätzli 5 (XP_037299529.1); *Bm*Spz5, *B. mori* spätzli 5 (XP_004924790.1); *Aa*Spz5like, *Anopheles albimanus* spätzle 5-like (XP_035790066.1); *Dme*Spz5, *D. melanogaster* spätzle5 (NP_647753.1); *Dma*Spz5, *Drosophila mauritiana* spätzle 5 (XP_033160799.1); *Ar*Spz5, *Athalia rosae* spätzli 5 (XP_012261687.1); *Pg*Spz5, *Pseudomyrmex gracilis* spätzle 5 isoform X3 (XP_020284715.1); and *Pv*Spz4, *Penaeus vannamei* spätzle 4 (ANJ04742.1).

### Analysis of *TmSpz5* Expression and Induction

The protocols for the developmental stage- and tissue-specific analyses have been reported previously (44, 45, 49). Briefly, total RNA was isolated from different developmental stages (eggs, young larvae (instars 10–12), late larvae (instars 14-15), pre-pupae, 1- to 7-day-old pupae, and 1- to 5-day-old adults) and tissues [integument, gut, fat bodies, Malpighian tubule (MT), hemocytes of last instar larvae and 5-day-old adults, and ovary and testis of 5-day-old adults] of *T. molitor*.

To analyze the induction of *TmSpz5*, suspensions containing 1 × 10^6^ cells/µl of *E. coli* and *S. aureus* and 5 × 10^4^ cells/μl of *C. albicans* were injected into *T. molitor* larvae at instars 10–12 (n = 20). PBS-injected *T. molitor* larvae were used as the control group. Samples were collected at 3, 6, 9, 12, and 24 h post-microbial challenge.

Total RNA was isolated using the Clear-S Total RNA Extraction Kit (Invirustech Co., Gwangju, South Korea) according to the manufacturer’s instructions. Then, 2 μg of total RNA was used as the template to synthesize cDNA using the Oligo (dT)12–18 primers under the following reaction conditions: 72°C for 5 min, 42°C for 1 h, and 94°C for 5 min. The MyGenie96 Thermal Block (Bioneer, Daejeon, Korea) and AccuPower^®^ RT PreMix (Bioneer) were used according to the manufacturer’s instructions. cDNA was stored at -20°C until further use.

Relative quantitative PCR (qRT-PCR) was performed using AccuPower^®^ 2X GreenStar qPCR Master Mix (Bioneer) with synthesized cDNAs and specific primers (*TmSpz5*_qPCR_Fw and *TmSpz5*_qPCR_Rv), as depicted in **Table 1**, with an initial denaturation of 95°C for 20 s, followed by 40 cycles at 95°C for 5 s and 60°C for 20 s. *T. molitor* ribosomal protein *L27a* (*TmL27a*) was used as an internal control, and the results were analyzed using the 2^-ΔΔCt^ method (50). The results are presented as means ± standard error (SE) of three biological replicates.

**Table 1:** Sequences of the primers used in this study.

Primer name	Sequence (5′–3′)
TmSpz5-qPCR-Fw	CAGTACGATGCACGAGAGGA
TmSpz5-qPCR-Rv	AACTGGGAAACCAGAACACG
TmSpz5-T7-Fw	TAATACGACTCACTATAGGGTCAGTACGATGCACGAGAGGA
TmSpz5-T7-Rv	TAATACGACTCACTATAGGGTAACTGGGAAACCAGAACACG
TmSpz5-cloning-Full ORF-Fw	CGCACATGTTGATGCATATTGAC
TmSpz5-cloning-Full ORF-Rv	TCTTTGTCTAACCGTTCGAGATG
TmL27a-qPCR-Fw	TCATCCTGAAGGCAAAGCTCCAGT
TmL27a-qPCR-Rv	AGGTTGGTTAGGCAGGCACCTTTA
dsEGFP-Fw	TAATACGACTCACTATAGGGTCGTAAACGGCCACAAGTTC
dsEGFP-Rv	TAATACGACTCACTATAGGGTTGCTCAGGTAGTGTTGTCG
TmTenecin-1_Fw	CAGCTGAAGAAATCGAACAAGG
TmTenecin-1_Rv	CAGACCCTCTTTCCGTTACAGT
TmTenecin-2_Fw	CAGCAAAACGGAGGATGGTC
TmTenecin-2_Rv	CGTTGAAATCGTGATCTTGTCC
TmTenecin-3_Fw	GATTTGCTTGATTCTGGTGGTC
TmTenecin-3_Rv	CTGATGGCCTCCTAAATGTCC
TmTenecin-4_Fw	GGACATTGAAGATCCAGGAAAG
TmTenecin-4_Rv	CGGTGTTCCTTATGTAGAGCTG
TmDefensin_Fw	AAATCGAACAAGGCCAACAC
TmDefencin_Rv	GCAAATGCAGACCCTCTTTC
TmDefencin-like_Fw	GCGATGCCTCATGAAGATGTAG
TmDefencin-like_Rv	CCAATGCAAACACATTCGTC
TmColeoptericinA_Fw	GGACAGAATGGTGGATGGTC
TmColeoptericinA_Rv	CTCCAACATTCCAGGTAGGC
TmColeoptericinB_Fw	CAGCTGTTGCCCACAAGTG
TmColeoptericinB_Rv	CTCAACGTTGGTCCTGGTGT
TmAttacin-1a_Fw	GAAACGAAATGGAAGGTGGA
TmAttacin-1a_Rv	TGCTTCGGCAGACAATACAG
TmAttacin-1b_Fw	GAGCTGTGAATGCAGGACAA
TmAttacin-1b_Rv	CCCTCTGATGAAACCTCCAA
TmAttacin-2_Fw	AACTGGGATATTCGCACGTC
TmAttacin-2_Rv	CCCTCCGAAATGTCTGTTGT
TmCecropin-2_Fw	TACTAGCAGCGCCAAAACCT
TmCecropin-2_Rv	CTGGAACATTAGGCGGAGAA
TmThaumatin-like protein-1_Fw	CTCAAAGGACACGCAGGACT
TmThaumatin-like protein-1_Rv	ACTTTGAGCTTCTCGGGACA
TmThaumatin-like protein-2_Fw	CCGTCTGGCTAGGAGTTCTG
TmThaumatin-like protein-2_Rv	ACTCCTCCAGCTCCGTTACA

Underline indicates T7 promotor sequence.

### RNA Interference

To synthesize the double-stranded RNA (dsRNA) of the *TmSpz5* gene, primers containing the T7 promoter sequence at their 5′ ends were designed using SnapDragon-Long dsRNA Design (**Table 1**). PCR was performed using AccuPower^®^ Pfu PCR PreMix with the *TmSpz5*_Fw and *TmSpz5*_Rv primers (**Table 1**) and according to the developmental expression pattern of *TmSpz5*, cDNA synthesized from pre-pupae (whole bodies) as a template under the following cycling conditions: an initial denaturation step at 94°C for 2 min followed by 35 cycles of denaturation at 94°C for 30 s, annealing at 53°C for 30 s, and extension at 72°C for 30 s, with a final extension step at 72°C for 5 min. PCR products were purified using the AccuPrep PCR Purification Kit (Bioneer), and dsRNA was synthesized using the AmpliScribe T7-Flash Transcription Kit (Epicentre Biotechnologies, Madison, WI, USA) according to the manufacturer’s instructions. After synthesis, the dsRNA was purified by precipitation with 5 M ammonium acetate and 80% ethanol, followed by quantification using an Epoch spectrophotometer (BioTek Instruments, Inc., Winooski, VT, USA). The dsRNA for enhanced green fluorescent protein (ds*EGFP*) was synthesized for use as a control and was stored at -20°C until use.

### Effect of *TmSpz5* Gene Silencing on Larval Mortality Against Microbial Challenge

To study the importance of *Tm*Spz5 in the *T. molitor* immune response, ds*TmSpz5* (1 µg/µl) was first injected into early-instar larvae (instars 10–12; n = 30) using disposable needles mounted onto a micro-applicator (Picospritzer III Micro Dispense System; Parker Hannifin, Hollis, NH, USA). An equal amount of ds*EGFP* was injected in the larvae at the same stage as the negative control. The efficiency of *TmSpz5* knockdown was evaluated by qRT-PCR, and over 86% knockdown was achieved at 4 days postinjection. The *TmSpz5*-silenced and ds*EGFP*-injected larval groups were challenged with *E. coli* (10^6^ cells/µl), *S. aureus* (10^6^ cells/µl), or *C. albicans* (5 × 10^4^ cells/µl) in triplicate experiments. The challenged larvae were maintained for 10 days, and the number of surviving larvae was recorded. The survival rates of *TmSpz5*-silenced larvae were compared with those of the control larvae. Relevant analysis was performed using Kaplan–Meier plots (51).

### Effect of ds*TmSpz5* on AMP Expression in Response to Microbial Challenge

To evaluate the functional properties of *TmSpz5* in the regulation of AMP gene expression in response to pathogens, RNAi was used for *TmSpz5* gene silencing, followed by the injection of larvae with *E. coli*, *S. aureus*, or *C. albicans*. ds*EGFP* and PBS were used as the negative and injection controls, respectively. At 24 h postinjection, the hemocytes, fat body, gut, and MTs were dissected, total RNA was extracted from each tissue, and cDNA was synthesized as described above. Next, qRT-PCR was performed with specific primers (**Table 1**) to analyze the temporal expression patterns of 14 AMP genes: *TmTenecin-1*, -*2*, -*3*, and -*4* (*TmTene1*, *2*, *3*, and *4*), *TmAttacin-1a*, -*1b*, and *-2* (*TmAtt1a*, *1b* and *2*), *TmDefensin* (*TmDef*), *TmDefensin-like* (*TmDef-like*), *TmColeoptericin-A* and -*B* (*TmColeA* and *B*), *TmCecropin-2* (*TmCec-2*), and *TmThaumatin like protein-1* and -*2* (*TmTLP1* and *2*).

### Effects of ds*TmSpz5* on NF-κB Gene Expression

To study the effects of ds*TmSpz5* on the expression of NF-κB genes, including *TmDorsal isoform X2* (*TmDorX2*) and *TmRelish* (*TmRel*), *TmSpz5* was silenced in early-instar larvae and *E. coli*, *S. aureus*, and *C. albicans* were injected at 4 days post-double-strand treatment. At 24 h after pathogen injection, the MTs, hemocytes, gut, and fat bodies were dissected. Total RNA extraction and cDNA synthesis were performed as described above.

### Effects of *TmSpz5* RNAi on Antimicrobial Activity Against *E. coli*

The AMPs and NF-κB gene expression patterns led us to perform colony-forming unit (CFU) assay to assess the *in vitro* AMP activity against Gram-negative bacteria. Therefore, *TmSpz5* dsRNA-treated young instar larvae of *T. molitor* were injected with *E. coli* (10^6^ cells). At 48 h post–pathogen injection, the hemolymph, midgut, hindgut, and Malpighian tubules were isolated in 100 μl 1× PBS. PBS and ds*EGFP* were injected as uninfected and dsRNA control groups, respectively. Tissue samples were homogenized and centrifuged at 15,000 rpm at 4°C for 10 min, and then the supernatants were boiled at 100°C for 10 min and centrifuged again at 15,000 rpm at 4°C for 10 min. Consequently, the protein content of extracted peptides has been measured by an Epoch machine and 50 ng of tissue samples was assayed with 10^6^ cells of *E. coli* in 1× PBS at 37°C for 2 h (52). Eventually, 2,000-fold serial dilutions were performed, and 100 μl of the resulting mixture was plated onto LB agar, followed by incubation at 37°C for 16 h. The colony numbers of assayed plates were then counted.

### Data Analysis

Statistical analyses were performed using SAS 9.4 (SAS Institute, Inc., Cary, NC, USA), and cumulative survival was analyzed by Tukey’s multiple-comparison test, with a significance level of *p* < 0.05. Fold change in expression of the AMP genes compared to the levels of the internal control (*TmL27a*) and external control (PBS) was calculated using the 2^-ΔΔCt^ method.

## Results

### *In Silico* Analysis of *TmSpz5*

**Figure 1:**
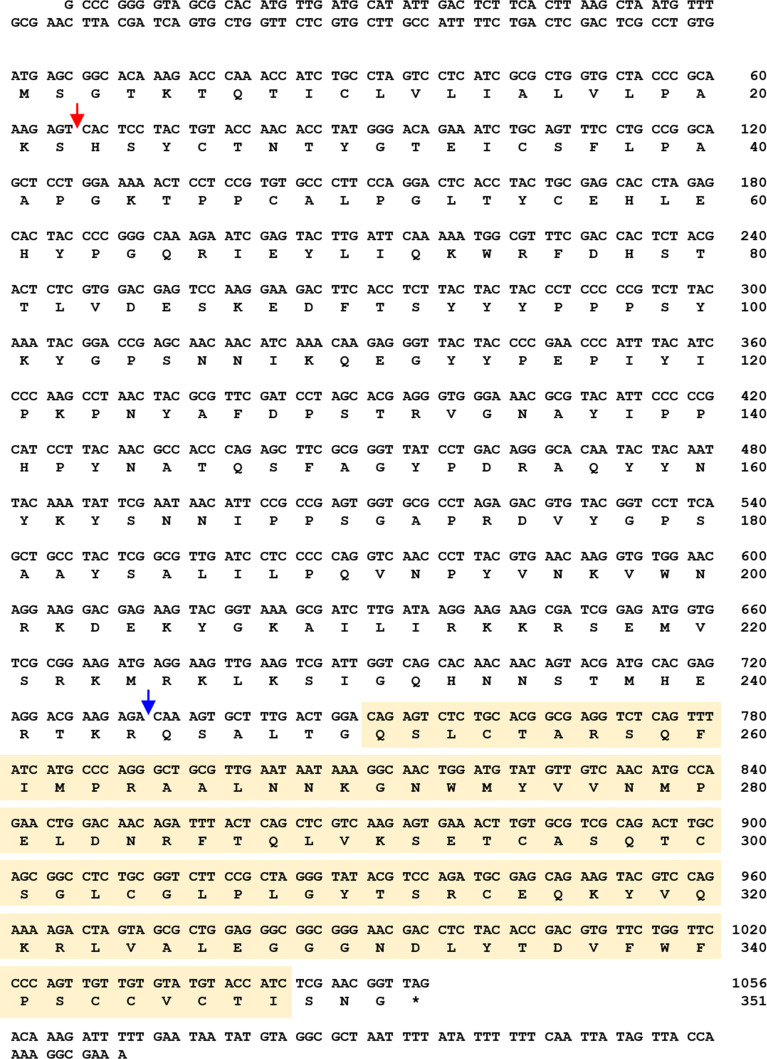
Nucleotide and deduced amino acid sequences of *T. molitor Spätzle5* (*TmSpz5*). The *Tm*Spz5 full-length open reading frame (ORF) consisted of 1,062 bp, encoding a polypeptide of 353 amino acid residues. The cystine-knot domain is shown in a yellow box, and the signal peptide region and cleavage site are indicated by red and blue arrows, respectively. The stop codon is shown with an asterisk.

**Figure 2:**
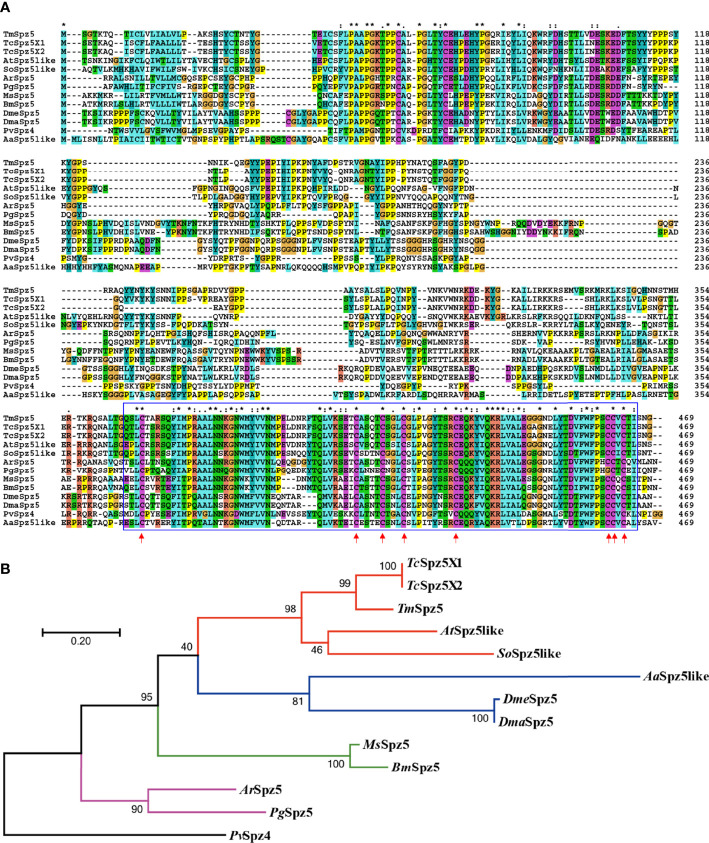
Multiple-sequence alignment **(A)** and phylogenetic analysis **(B)** of *T. molitor* Spätzle5 (*Tm*Spz5). A domain analysis was performed using ClustalX2, and the phylogenetic tree was constructed using MEGA7 with the maximum likelihood method and 1,000 bootstrap replicates (where numbers at nodes indicate bootstrap support). The representative Spätzle 5 protein sequences showed high homology at the conserved domains marked in blue boxes, and the red arrows indicate conserved cystine-knot domain scores between groups. A neighbor-joining (NJ) tree was constructed based on the protein sequences of *Tc*Spz5, *Tribolium castaneum* spätzle 5 isoform X1 (XP_008193940.1); *Tc*Spz5, *Tribolium castaneum* spätzle 5 isoform X2 (XP_015836109.1); *At*Spz5like, *A*. *tumida* spätzle 5-like (XP_019879590.1); *So*Spz5like, *S. oryzae* spätzle 5-like (XP_030767938.1); *Ms*Spz5, *M. sexta* spätzle 5 (XP_037299529.1); *Bm*Spz5, *B*. *mori* spätzle 5 (XP_004924790.1); *Aa*Spz5like, *A*. *albimanus* spätzle 5-like (XP_035790066.1); *Dme*Spz5, *D*. *melanogaster* spaetzle5 (NP_647753.1); *Dma*Spz5, *D*. *mauritiana* spätzle 5 (XP_033160799.1); *Ar*Spz5, *A*. *rosae* spätzle 5 (XP_012261687.1); *Pg*Spz5, *P. gracilis* spätzle 5 isoform X3 (XP_020284715.1); and *Pv*Spz4, *P. vannamei* spätzle 4 (ANJ04742.1) which was used as the outgroup. Colored lines indicate different insect orders; red: Coleopteran, green: Lepidopteran, blue: Dipteran, purple: Hymenopteran. *Pv*Spz4, illustrated black, belongs to the Crustacean class.

To acquire the full-length cDNA sequence of *TmSpz5*, a local blast search of the *T. molitor* RNAseq database was performed using the *T. castaneum* Spätzle5 protein sequence as the query. The *TmSpz5* full-length ORF consisted of 1,062 bp, encoding a polypeptide of 353 amino acid residues (**Figure 1**). As determined using InterProScan, the *Tm*Spz5 amino acid sequence contained a cystine-knot domain at the C-terminus (which binds to the Toll receptor), one cleavage site predicted to be processed by SPE, and a predicted signal peptide at the N-terminus (**Figure 1**). Additionally, the conserved domains in *Tm*Spz*5* were compared at the amino acid level using ClustalX 2.1 and multiple-sequence alignment. *Tm*Spz5 sequences were conserved at the protein level among insect species (**Figure 2A**). A phylogenetic analysis illustrated that *Tm*Spätzle5 in the order Coleoptera formed a group with other isoforms of Spätzle5 from *T. castaneum* (**Figure 2B**).

### Temporal and Spatial Expression of *TmSpz5*

**Figure 3:**
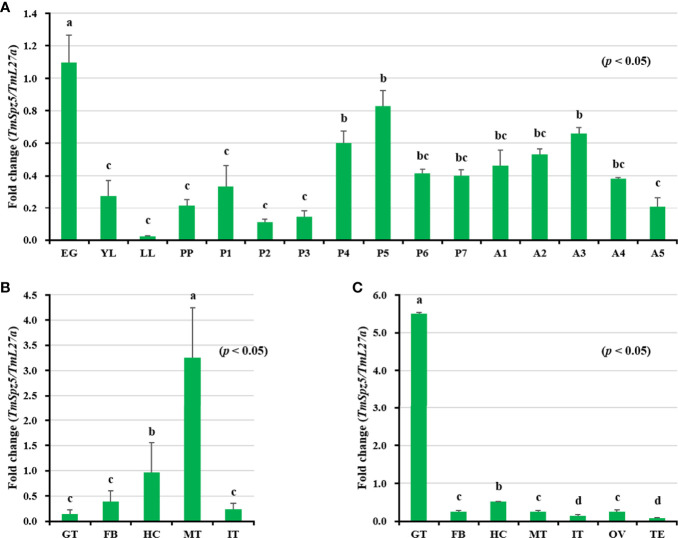
Developmental stage- and tissue-specific expression patterns of *TmSpz5* measured by qRT-PCR. **(A)** Relative *TmSpz5* mRNA levels in eggs (EG), young larvae (YL), late-instar larvae (LL), pre-pupae (PP), 1- to 7-day-old pupae (P1–P7), and 1- to 5-day-old adults (A1–A5) are illustrated. Expression levels were the highest in the eggs and the pupae. The mRNA levels decreased in the larval stage and were lowest in the late larval stage. Increases in *TmSpz5* transcript levels were detected during molting and in each ecdysis with a gradual decrease across each individual stage. *TmSpz5* tissue expression patterns in late instar larvae **(B)** and adults **(C)** were also examined. Total RNA was extracted from different tissues, including the integument (IT), Malpighian tubule (MT), gut (GT), hemocytes (HC), and fat bodies (FB) of late instar larvae and the integument (IT), Malpighian tubule (MT), gut (GT), hemocytes (HC), fat bodies (FB), ovary (OV), and testis (TE) of 5-day-old adults. Total RNA was isolated from 20 mealworms and *T. molitor* 60S ribosomal protein 27a (*TmL27a*) primers were used as internal control (N = 3). One-way ANOVA and Tukey’s multiple-range test at a 95% confidence level were used for comparisons. Bars with the same letter are not significantly different by Tukey’s multiple-range test (*p* < 0.05).

*TmSpz5* mRNA expression patterns were evaluated by qRT-PCR at different developmental stages and in various tissues in larvae and adults. *TmSpz5* was observed at essentially all developmental stages (**Figure 3A**). However, the highest expression levels were seen in embryos and pupae. The mRNA levels decreased at the larval stage, and in late larvae, it shows the lowest expression. We observed fluctuations in the expression pattern during pupal stages with a plateau phase in late pupae. Overall, increased *TmSpz5* mRNA levels were observed during molting and each ecdysis, with a gradual fall across each individual stage.

With respect to tissue expression patterns (**Figures 3B, C** **)**, *TmSpz5* expression levels were highest in MTs, followed by (in decreasing order) the hemocytes, fat bodies, integument, and gut in larvae. Contrarily, in adults, the mRNA expression of *TmSpz5* was low in MTs and highest in the gut.

### Patterns of *TmSpz5* Induction

**Figure 4:**
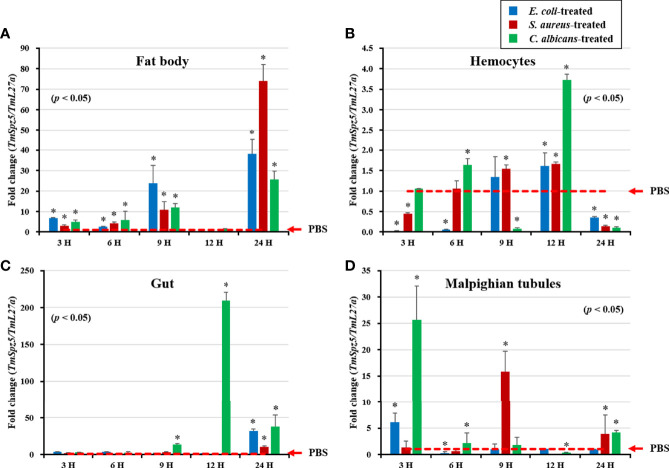
Temporal expression patterns of *TmSpz5* in immune-challenged *T. molitor* larvae. Levels of *TmSpz5* mRNA in the fat bodies **(A)**, hemocytes **(B)**, gut **(C)**, and Malpighian tubules **(D)** were examined by qRT-PCR 3, 6, 9, 12, and 24 h after infection with *E*. *coli* (10^6^ cells/µl)*, S. aureus* (10^6^ cells/µl), and *C*. *albicans* (5 × 10^4^ cells/µl). *TmSpz5* expression was highly induced in the presence of *C. albicans* and *S. aureus* in various tissues. PBS was used as an injection control, and *T. molitor* 60S ribosomal protein 27a (*TmL27a*) primers were used as internal control (n = 3). Asterisks indicate significant differences between infected and PBS-injected larval groups by Student’s *t*-test (*p* < 0.05). Vertical bars indicate means ± SD (n = 20).

*TmSpz5* expression in immune-challenged *T. molitor* larvae was examined after *E. coli*, *S. aureus*, and *C. albicans* injections (**Figure 4**), using PBS injection as the control. Four tissues, including the fat bodies (**Figure 4A**), hemocytes (**Figure 4B**), gut (**Figure 4C**), and MTs (**Figure 4D**), were collected at 3, 6, 9, 12, and 24 h post-pathogen injection for total RNA extraction. *TmSpz5* expression was considerably upregulated in response to bacterial and fungal infections. *TmSpz5* expression varied in tissue- and time-dependent manners. The highest expression levels were seen in the gut at 12 and 24 h and in the fat bodies at 9 and 24 h after infection (in that order), in response to all three pathogens. Of note, in the fat bodies, the expression of *TmSpz5* was lowest at 12 h, possibly due to fluctuations in mRNA expression after infection as also reported earlier (35, 44, 49, 53). In MTs, there was also a noticeable upregulation in response to *C. albicans* and *E. coli* at 3 h post injection and in response to *S. aureus* at 9 h postinjection. *C. albicans* also induced *TmSpz5* expression in the hemocytes at 12 h postinjection.

### Effect of *TmSpz5* RNAi on *T. molitor* Survival

**Figure 5:**
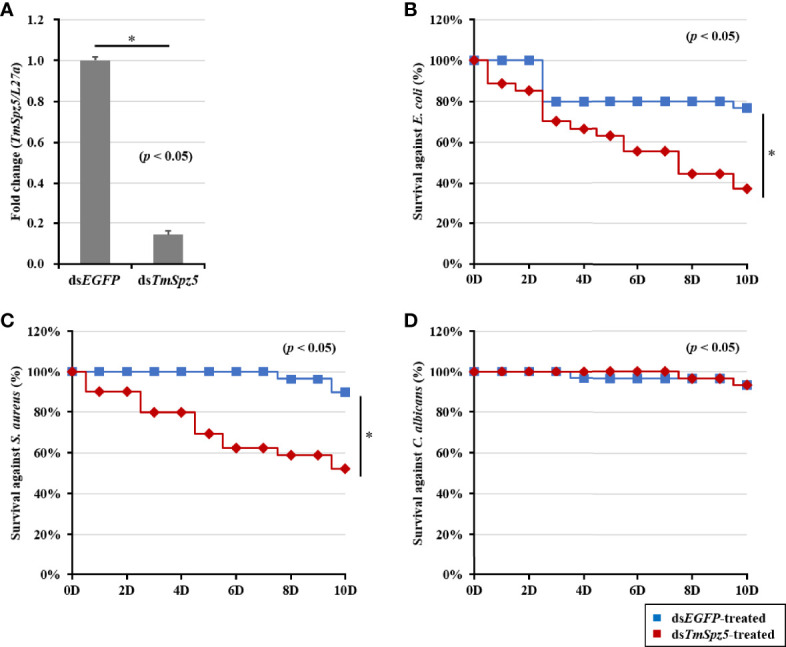
Effect of *TmSpz5* gene silencing on the survival of *T. molitor* larvae. The silencing efficiency of ds*TmSpz5* was measured by qRT-PCR at 4 days postinjection **(A)**. *TmSpz5*-silenced larvae were injected with *E*. *coli* **(B)**, *S. aureus* **(C)**, and *C*. *albicans* **(D)**, and survival rates were studied over 10 days post-pathogen injection (n = 10 per group). Larval survival rates at 10 days post-microbial injection were 33% after *E. coli* injection, 58% after *S. aureus* injection, and 90% after *C*. *albicans* injection compared with the levels in the ds*EGFP-*injected control group. The data are reported as averages of three biologically independent replicates. Asterisks indicate significant differences between ds*TmSpz5*- and ds*EGFP*-injected groups. The survival analysis was performed using Kaplan–Meier plots (log-rank chi-squared test; *p < 0.05).

Considering our observation that *TmSpz5* expression is induced by different pathogens, we further examined the survival rate of *TmSpz5*-silenced larvae using the RNAi technique. *TmSpz5* mRNA levels were decreased by 86% 4 days after ds*TmSpz5* injection (**Figure 5A**), confirming the efficiency of the RNAi.

Subsequent to confirmation of RNAi efficiency, pathogens of interest were injected. Survival rates of *TmSpz5*-silenced larvae were then evaluated over 10 days following microbial infection. ds*EGFP* was used as the control group for ds*TmSpz5.* PBS-injected larvae showed no statistically significant differences in survival between the ds*TmSpz5* and ds*EGFP* groups (data not shown). ds*TmSpz5* larvae showed considerable reductions in survival in response to *E. coli* and *S. aureus* (survival rates of approximately 33% and 58%, respectively) (**Figures 5B, C** **)**. Interestingly, *C. albicans*-injected larvae showed similar survival rates to those of the PBS group (**Figure 5D**).

### Effect of *TmSpz5* Gene Silencing on Antimicrobial Peptide Production

The survival analysis indicated that *TmSpz5* gene silencing accelerated the vulnerability of larvae challenged with *E. coli* and *S. aureus*, but not *C. albicans*. We further evaluated the induction of AMPs following challenge with *E. coli*, *S. aureus*, and *C. albicans* in *TmSpz5-*silenced *T. molitor* larvae. In particular, we knocked down *TmSpz5* and evaluated the levels of 14 AMP genes 24 h after the microbial challenge.

**Figure 6:**
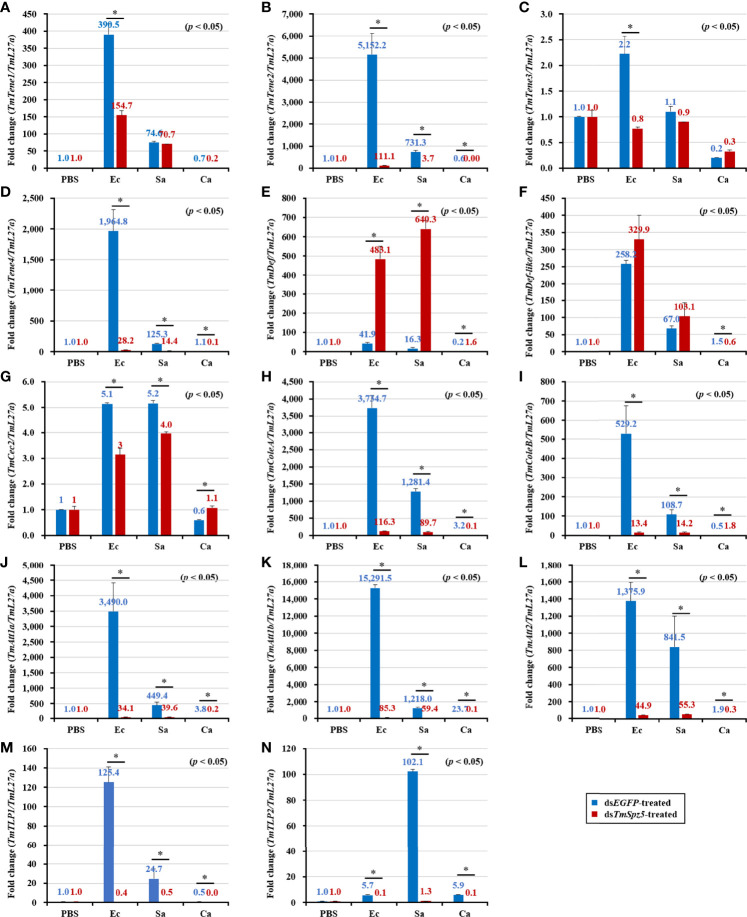
Induction of 14 AMP genes in the Malpighian tubules of *TmSpz5*-silenced *T. molitor* larvae infected with *E. coli* (Ec)*, S. aureus* (Sa), and *C. albicans* (Ca) using PBS as control. At 24 h after microbial injection, AMP genes, including *TmTene1* **(A)**, *TmTene2* **(B)**, *TmTene3* **(C)**, *TmTene4* **(D)**, *TmDef* **(E)**, *TmDef-like* **(F)**, *TmCec2* **(G)**, *TmColeA* **(H)**, *TmColeB* **(I)**, *TmAtt1a* **(J)**, *TmAtt1b* **(K)**, *TmAtt2* **(L)**, *TmTLP1* **(M)**, and *TmTLP2* **(N)** were examined by qPCR using ds*EGFP* as a knockdown control and *T. molitor ribosomal protein* (*TmL27a*) as an internal control. All experiments were performed in triplicate. Asterisks indicate significant differences between ds*TmSpz5*- and ds*EGFP*-treated groups determined by Student’s *t*-test (*p* < 0.05).

**Figure 7:**
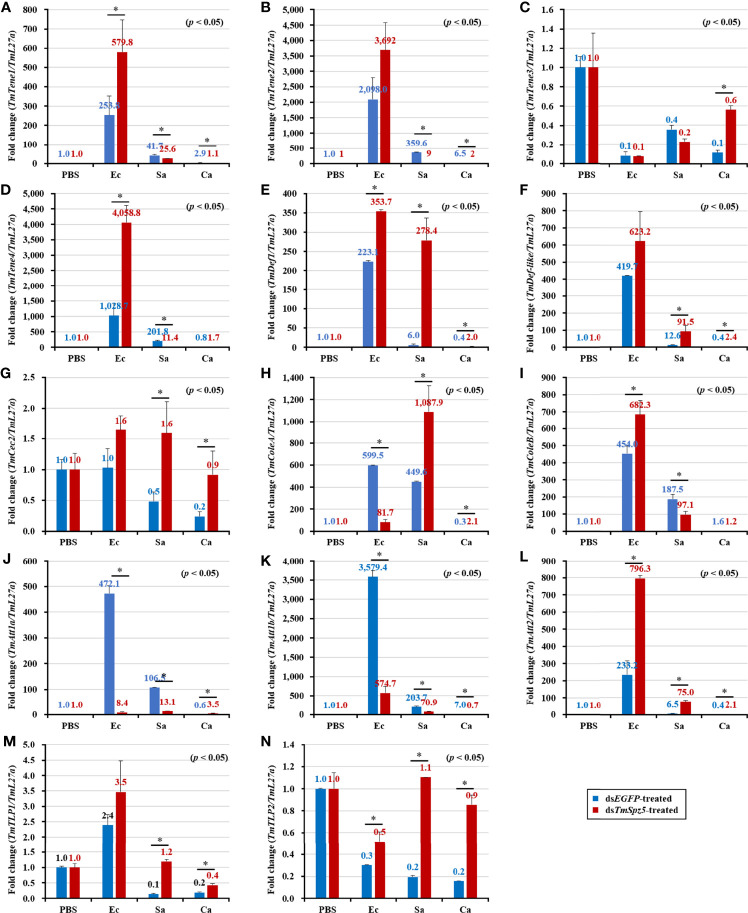
Effect of *TmSpz5* gene silencing on antimicrobial peptide (AMP) gene expression levels in the *T. molitor* gut. Four days after *TmSpz5* RNAi treatment, pathogens including *E. coli* (Ec), *S. aureus* (Sa), and *C. albicans* (Ca) and PBS as control were injected. Levels of the AMP genes, including *TmTene1* **(A)**, *TmTene2* **(B)**, *TmTene3* **(C)**, *TmTene4* **(D)**, *TmDef* **(E)**, *TmDef-like* **(F)**, *TmCec2* **(G)**, *TmColeA* **(H)**, *TmColeB* **(I)**, *TmAtt1a* **(J)**, *TmAtt1b* **(K)**, *TmAtt2* **(L)**, *TmTLP1* **(M)**, and *TmTLP2* **(N)** were evaluated by qRT-PCR at 24 h post-microbial injection. ds*EGFP* was injected as a negative control, and *TmL27a* expression was evaluated as an internal control. All experiments were performed in triplicate. Asterisks indicate significant differences between ds*TmSpz5*- and ds*EGFP*-treated groups when compared using Student’s *t*-test (*p* < 0.05).

**Figure 8:**
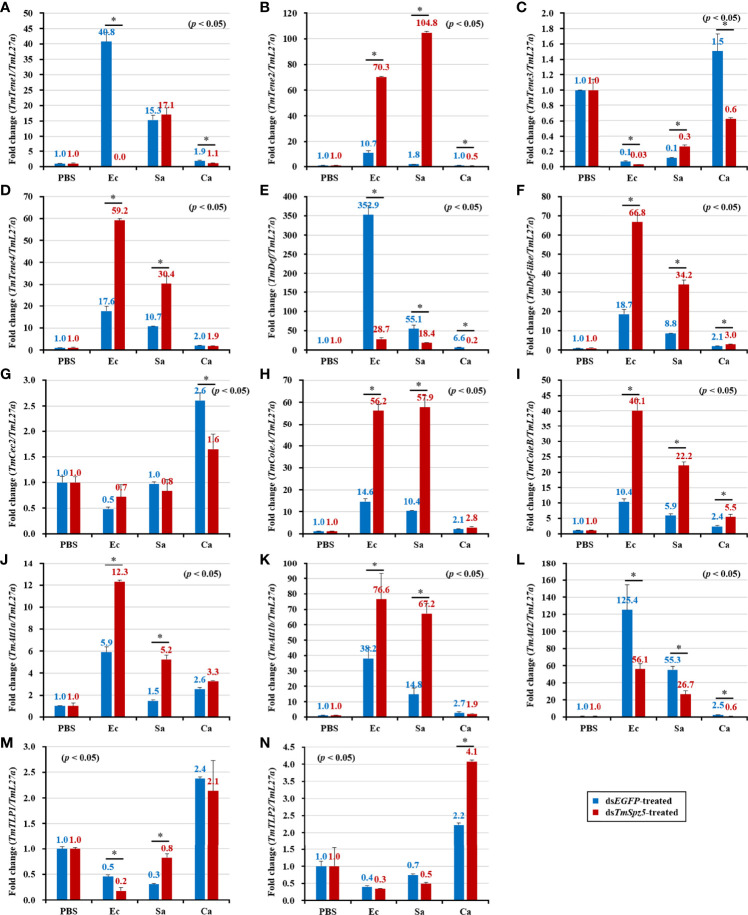
The mRNA expression levels of 14 antimicrobial peptide (AMP) genes after *TmSpz5* gene silencing in the hemocytes of *T. molitor*. Four days after *TmSpz5* dsRNA treatment, *E. coli* (Ec), *S. aureus* (Sa), and *C. albicans* (Ca) and PBS as a control were injected. At 24 h after injecting the microbes, the expression levels of *TmTene1* **(A)**, *TmTene2* **(B)**, *TmTene3* **(C)**, *TmTene4* **(D)**, *TmDef* **(E)**, *TmDef-like* **(F)**, *TmCec2* **(G)**, *TmColeA* **(H)**, *TmColeB* **(I)**, *TmAtt1a* **(J)**, *TmAtt1b* **(K)**, *TmAtt2* **(L)**, *TmTLP1* **(M)**, and *TmTLP2* **(N)** were evaluated by qRT-PCR. ds*EGFP* was injected as a negative control, and *TmL27a* expression was measured as an internal control. All experiments were performed in triplicate. Asterisks indicate significant differences between ds*TmSpz5-* and ds*EGFP*-treated groups when compared using Student’s *t*-test (*p* < 0.05).

**Figure 9:**
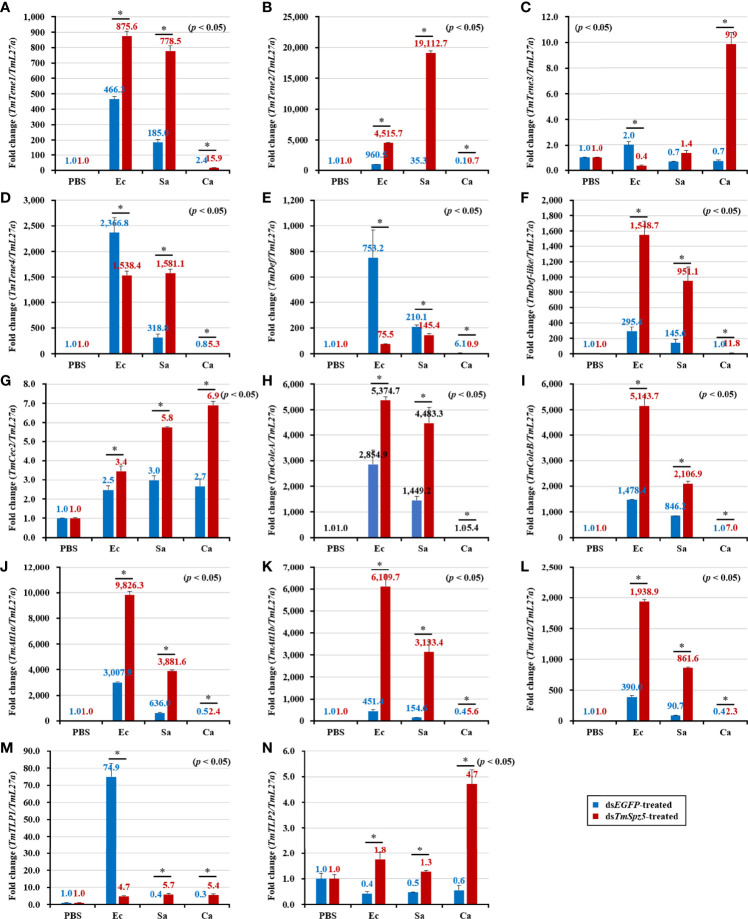
Antimicrobial peptide expression patterns in the fat bodies of ds*TmSpz5-*treated *T. molitor* larvae in response to microbial challenge. At 24 h post-infection by *E*. *coli* (Ec), *S. aureus* (Sa), and *C. albicans* (Ca), the mRNA levels of *TmTene1* **(A)**, *TmTene2* **(B)**, *TmTene3* **(C)**, *TmTene4* **(D)**, *TmDef* **(E)**, *TmDef-like* **(F)**, *TmCec2* **(G)**, *TmColeA* **(H)**, *TmColeB* **(I)**, *TmAtt1a* **(J)**, *TmAtt1b* **(K)**, *TmAtt2* **(L)**, *TmTLP1* **(M)**, and *TmTLP2* **(N)** were evaluated by qRT-PCR. PBS was administered to the non-infected control group. ds*EGFP* was injected as a negative control, and *TmL27a* expression was measured as an internal control. All experiments were performed in triplicate. Asterisks indicate significant differences between ds*TmSpz5*- and ds*EGFP*-treated groups determined using Student’s t-test (*p* < 0.05).

According to the results of the survival analysis, we expected *TmSpz5* silencing to lead to AMP downregulation in response to *E. coli* and *S. aureus.* Our data illustrated that following confirmation of the *TmSpz5* knockdown efficiency (**Supplementary Figure 1**), 10 out of 14 AMP genes were significantly downregulated in the MTs of *TmSpz5-*silenced larvae after *E. coli* and *S. aureus* injections but not after fungal infection. In particular, the *E. coli* challenge resulted in reductions in the levels of *TmTene1*, *TmTene2*, *TmTene3*, *TmTene4*, *TmColeA*, *TmColeB*, *TmAtt1a*, *TmAtt1b*, *TmAtt2*, *TmTLP1*, and *TmTLP2* and the *S. aureus* challenge resulted in substantial reductions in the levels of *TmTene2*, *TmTene4*, *TmColeA*, *TmColeB*, *TmAtt1a*, *TmAtt1b*, *TmAtt2*, *TmTLP1*, and *TmTLP2* (**Figure 6**). In the gut, silencing of *TmSpz5* suppressed the *E. coli-*induced upregulation of *TmColeA*, *TmAtt1a*, and *TmAtt1b* as well as the S. *aureus*-induced regulation of *TmTene2*, *TmTene4*, *TmColeB*, *TmAtt1a*, and *TmAtt1b* (**Figures 7B, D, H–K**). In the hemocytes, *TmTene1*, *TmDef*, and *TmAtt2* were downregulated in response to *E. coli* and *TmDef* and *TmAtt2* were downregulated in response to *S. aureus* (**Figures 8A, E, L**). Moreover, in response to *C.albicans*, mRNA levels of TmTen3 and TmCec2 were downregulated (**Figures 8C, G**). In the fat bodies, only the levels of *TmTene4*, *TmDef*, and *TmTLP1* were reduced in response to *E. coli* infection (**Figures 9D, E, M**). Surprisingly, ds*TmSpz5* elevated the mRNA levels of some AMPs in response to pathogens in all dissected tissues, particularly the levels of the *Cecropin*, *Attacin*, and *Tencin* families in the gut, fat bodies, and hemocytes (**Figures 7A, C, E–G**, **8B, D, F**, and **9A–C, F–L, N**). Finally, mRNA levels of almost all AMPs did not differ between the ds*TmSpz5* group and the control group in response to *C. albicans.*

**Figure 10:**
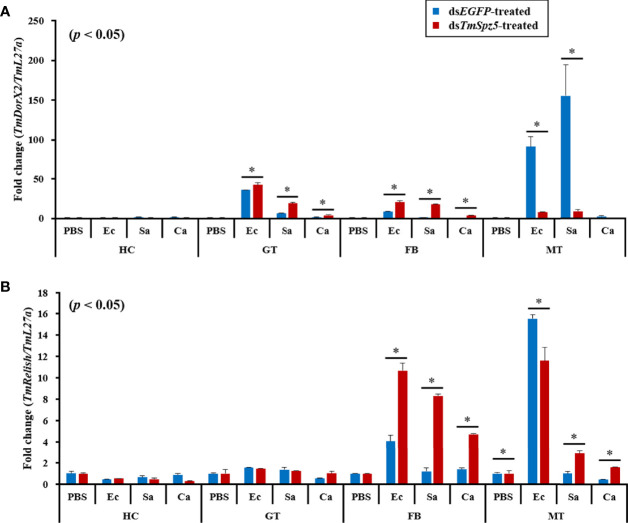
Effect of *Tmspz5* gene silencing on NF-κB gene expression patterns. ds*Tmspz5*-treated *T. molitor* larvae were infected with *E*. *coli*, *S. aureus* and *C. albicans* and 24 h post-pathogen injection, mRNA levels of NF-κB genes, including *TmDorX2* **(A)** and *TmRel* **(B)**, were measured by RT-qPCR. EGFP dsRNA was assessed as a negative control and *T. molitor ribosomal protein* (*TmL27a*) was used as an internal control. All experiments were performed in triplicate. Asterisks indicate significant differences between ds*TmSpz5*- and ds*EGFP*-treated groups determined using Student’s *t*-test (*p* < 0.05).

Following the same protocol used to evaluate the expression of AMP genes following knockdown, the NF-κB pathway genes *TmDorX2* and *TmRelish* were examined (**Figure 10**). ds*TmSpz5* considerably depleted *TmDorX2* expression levels in MTs following *E. coli* and *S. aureus* infection (**Figure 10A**). A less substantial reduction in *TmRelish* expression was observed in MTs (**Figure 10B**). Moreover, following the microbial challenge, *TmDorX2* was upregulated in the fat bodies and gut and *TmRelish* was upregulated in the fat bodies.

### Loss of Antimicrobial Activity in ds*TmSpz5*-Treated Larvae

**Figure 11:**
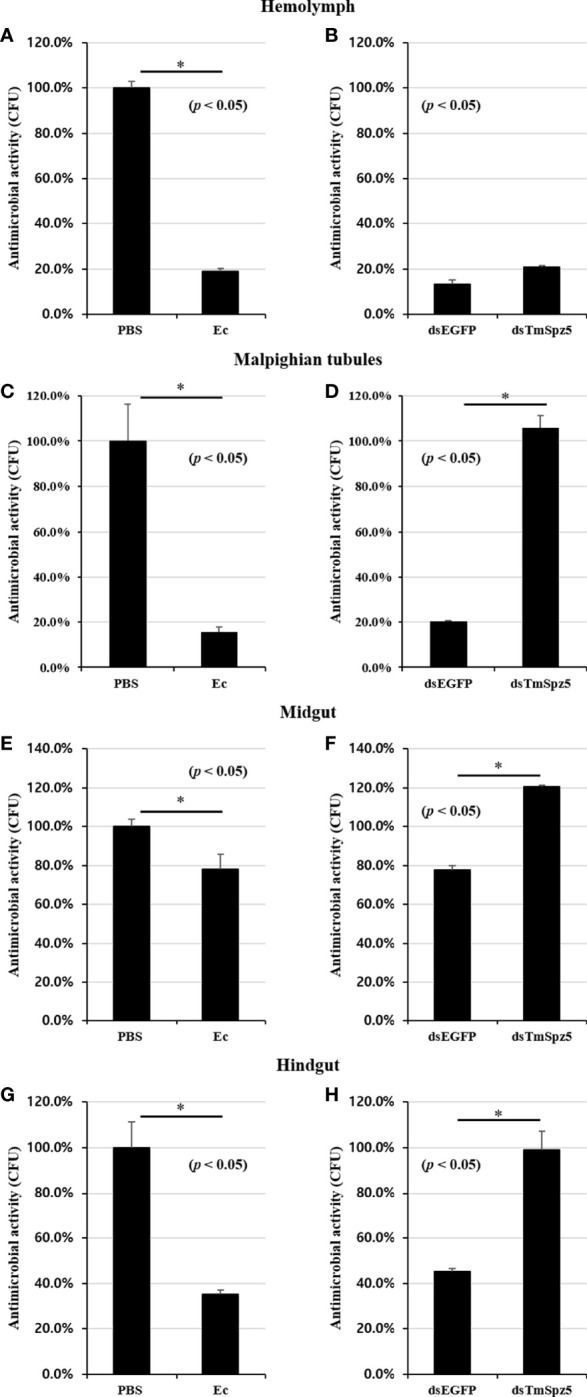
Antimicrobial activity against *E. coli* in *TmSpz5*-silenced larvae hemolymph, Malpighian tubules, midgut, and hindgut by CFU assay. **(A, C, E, G)** Antimicrobial activity evoked by *E*. *coli* (Ec) (10^6^ cells/μl) elicitation. PBS-injected *T. molitor* was used as a negative control (Cont). *E*. *coli*-injected *T. molitor* hemolymph and Malpighian tubules had higher antimicrobial activity compared with control group **(A, C)**. **(B, D, F, H)** *E*. *coli* (10^6^ cells/μl) was injected into ds*TmSpz5*-treated *T. molitor* larvae. ds*EGFP*-treated larvae were used as a negative control. The result shows that the antimicrobial activity was decreased by treatment of ds*Spz5* compared with the ds*EGFP*-treated group majorly in Malpighian tubules **(D)**, hindgut **(H)**, and midgut **(F)** in a depleting manner. *E. coli* proliferation remained indifferent in the ds*TmSpz5*-treated group compared with ds*EGFP*-treated larvae in hemolymph **(B)**. Asterisks indicate significant differences between ds*TmSpz5-* and dsEGFP-injected groups.

The AMP assay result clearly demonstrated that *E. coli* and *S. aureus* infection in ds*TmSpz5*-treated larvae induced AMP expression significantly in Malpighian tubules and partially in the gut. Thus, we examined whether this suppression would affect bacterial growth in the hemolymph, MTs, midgut, and hindgut by CFU assay. Following ds*EGFP* and ds*TmSpz5* injection, larvae were exposed to *E. coli*, and the aforementioned tissues were dissected 48 h postinfection and cultured with *E. coli* on LB agar plates. Elevated antimicrobial activity was observed in all dissected tissues in *E. coli*-injected larvae compared with the PBS-injected group (**Figures 11A, C, E, G** **)**. Moreover, it was found that *E. coli* growth was hindered in the ds*EGFP*-injected gut, compared to the ds*TmSpz5*-injected larvae in the MTs, hindgut, and midgut (in decreasing order) (**Figures 11D, F, H** **)**. In contrast, in the hemolymph, no significant difference in proliferation inhibition was observed between the ds*EGFP*- and ds*TmSpz5*-injected larvae (**Figure 11B**). These results imply that the effect of *TmSpz5* knockdown on AMP gene depletion in MTs causes suppressed antimicrobial activity against Gram-negative bacteria. Additionally, while antimicrobial activity in hemolymph remained indifferent, downregulation of AMP genes in MTs subsequent to *TmSpz5* knockdown exhibits reduced antimicrobial activity in the hindgut.

## Discussion

*Drosophila* is one of the most potent genetic model systems for characterization of the Toll and Imd signaling pathways (24, 54, 55). Nevertheless, the focus on this model limits our understanding of the biochemical mechanisms of the Toll proteolytic cascade. For instance, the activation protocol (i.e., developmental factors or infection) influences pathway activity, making it difficult to comprehensively characterize the underlying mechanisms (12, 31).

*T. molitor* Toll signaling activation by Gram-positive bacteria or fungi, its compartments, and its relevant AMPs have been well elucidated (24). Surprisingly, similar to Lys-type PGN, *Tm*PGRP-SA can recognize polymeric DAP-type PGN of Gram-negative bacteria, subsequently leading to activation of a three-step proteolytic cascade and the production of mature Spätzle (53, 56).

During *Drosophila* developmental stages, expression of the Spätzle gene is regulated by hormonal alteration. Radio-immunoassays have illustrated that ecdysone activity is high during prepupal and pupal stages (57). Likewise, cross talk between the steroid hormone 20-hydroxyecdysone (20E) and immune-regulatory genes in *Drosophila* has been reported (57, 58). Additionally, *Drosophila* MTs do not undergo histological alterations during pupal metamorphosis and therefore play an important role in innate immunity during this process (59, 60). Our developmental stage- and tissue-specific gene expression data revealed that *TmSpz5* levels are high during the embryonic stage as well as at each ecdysis, consequently increasing susceptibility to possible attacks, showing that *TmSpz5* contributes to both insect dorso-ventral axis formation during development and immune responses, respectively (11). The fact that *TmSpz5* expression is the highest in larval MTs and in the adult gut supports its important role in epithelial defense organs.

Toll signaling is activated upon the recognition of Gram-positive bacteria and fungi by the cleavage of the cytokine-like polypeptide Spätzle (2, 6, 27). We detected a high and early expression of *TmSpz5* following *C. albicans* and *S. aureus* challenge in descending order in all dissected tissues. The observed *TmSpz5* expression in response to *E. coli* infection in MTs provides evidence for cross talk between the Toll and Imd signaling pathways. The unexpected results of the survival analysis demonstrated the importance of *TmSpz5* in *T. molitor* immunity against *E. coli* and *S. aureus*, but not *C. albicans*. Consistently, *TmSpz5* silencing leads to *T. molitor* vulnerability toward *E. coli* and *S. aureus* by decreasing AMP production in the presence of pathogens. Our results were predominantly consistent with those of previous studies on AMP production after treatment with ds*TmSpz5.* In *Drosophila*, attacin, diptericin, cecropin, and drosocin are active against Gram-negative bacteria, and metchnikowin and defensin act against Gram-positive bacteria (59, 61–65). In this study, *TmCecropin-2* was also induced by Gram-positive bacteria and fungi. Surprisingly, the elevated mRNA levels of some AMPs in various tissues may suggest that there are alternative mechanisms to regulate gene expression. As it has been demonstrated previously, different *T. molitor Späzle* RNAi treatments (*TmSpz4*, *TmSpz6*, *TmSpzlike*) resulted in an increased expression of AMPs following microbial challenges (44, 45, 49). Additionally, the monomeric DAP-type peptidoglycan of Gram-negative bacteria activates *Tm*IMD protein which triggers the expression of nine AMP genes (66). Likely, results of this study propose a possibility that the effect of *TmSpz5* RNAi leads to the overexpression of other *Späzle* genes with a similar function. Overexpression of some AMP genes, mostly in hemolymph and fat bodies, maintains homeostasis. Moreover, other signaling pathways such as Imd can trigger an elevated expression of AMPs (35). Since the Imd signaling pathway has not been fully clarified, further studies regarding possible synergistic effects on induction of different AMPs are required to have a crystal understanding of Toll and Imd pathway association with regulation of AMP genes. Furthermore, the lack of change in mRNA levels of most AMPs in the ds*TmSpz5* group in response to *C. albicans* appeared to be inconsistent with the induction data.

NF-κB family members in *Drosophila*, activated by the Toll and Imd pathways, regulate the expression of AMP genes (27). The Toll signaling pathway mediates activation of the transcription factors Dorsal and Dif and is predominantly actuated by the detection of Gram-positive bacteria and fungi (6, 40, 67, 68). In contrast, Gram-negative bacteria activate the Imd pathway, which triggers the NF-κB transcription factor Relish (6, 43, 69). In agreement with the AMP expression results, *TmDorX2* was significantly suppressed in the MTs of *TmSpz5*-silenced larvae following challenges with *E. coli* and *S. aureus*, indicating that *TmSpz5* is involved in regulating the expression of *TmDorX2.*

With respect to antimicrobial activity, AMPs extracted from all tissues except the hemolymph effectively inhibited *E. coli* growth. The effective inhibition of bacterial proliferation in the MTs and hindgut were consistent with the AMP mRNA expression and NF-κB results, suggesting that *TmSpz5* acts as an immune component in the MTs and subsequently the hindgut. Further investigations are needed to verify these results.

*Drosophila* fat bodies are considered as the insect equivalent of the mammalian liver and are the main AMP-producing tissues, allowing an effective response to infection (6). Epithelial cells in the gut, MTs, genital tract, and trachea play important roles in systemic immunity by mediating the local response to invaders (70, 71). These epithelial tissues constitute the first line of defense toward possible invaders, and if pathogens invade these barriers, cellular and humoral immunity is induced (60, 71). Insect MTs form by hindgut–midgut joint invagination, and thus its secretions and hemolymph waste products are constantly transported to the hindgut (72). Thereupon, *Drosophila* MTs have osmoregulatory activity function as detoxification compartments in the hemolymph, acting as major innate immune organs (73). They are able to recognize pathogens and induce the production of high levels of AMPs (59, 60). They do not endure metamorphosis caused by ecdysone induction and are conveyed from larvae to adults. PGRP-LC expression is elevated by MT ecdysone production and has a marked effect on boosting host immunity (60). Additionally, PGRP-LE and PGRP-SC1 are immune elements predominantly functioning in the posterior midgut and anterior hindgut (74).

**Figure 12:**
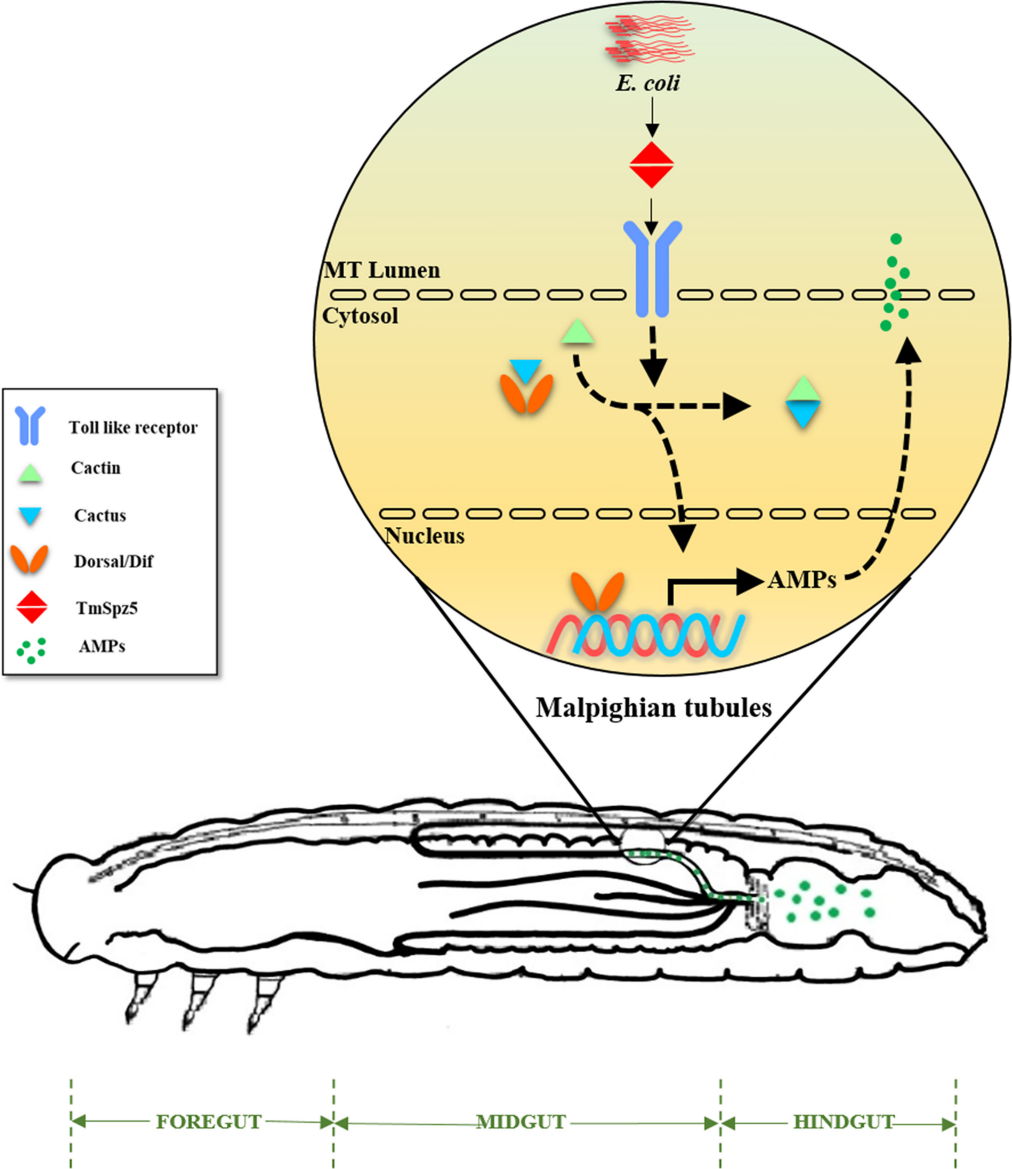
A schematic summary of *TmSpz5* positive regulation in antimicrobial peptide production in Malpighian tubules (MTs) of bacterial infected larvae. 10 AMP-encoding genes including *TmTen-1*, *TmTen-2*, *TmTen-4*, *TmColA*, *TmColB*, *TmAtt-1a*, *TmAtt-1b*, *TmAtt-2*, *TmTLP-1*, and *TmTLP-2* are positively regulated by *TmSpz5* upon bacterial infections in MTs and produced peptides along with rest of tubules content, eventually flow to hindgut.

Our results show that MTs are critical immune organs in *T. molitor*, as has been observed in *Drosophila.* The radical shrinkage of the expression of almost all AMP genes and *TmDorX2* in the MTs of *TmSpz5*-silenced larvae following *E. coli* infection suggests that DAP-type PGN is recognized by PRRs and the relevant proteolytic cascade leads to the activation of mature *Tm*Spz5. Consequently, activated *Tm*Spz5 binds to the Toll receptor and positively regulates the expression of the NF-κB response elements and AMP genes. In contrast to the lack of change in AMP expression in the gut of *Tm*Spz5 knockdown larvae, the CFU results not only demonstrate the pivotal role of *Tm*Spz5 in antibacterial activity of MT AMPs but also show that these AMPs act as hindgut disinfectants (**Figure 12** **).**

Our molecular analyses deepen our current knowledge of *T. molitor* immunity. Notably, the role of MTs in the innate immunity of *T. molitor* against the Gram-negative bacteria, *E. coli*, supports the results of previous studies, showing that polymeric DAP-type PG can be sensed by PGRP-SA, and Toll pathway activation leads to *Tm*Spz5 cleavage and AMP production. A comprehensive understanding of these proteolytic cascades could provide a basis for the development of diagnostic kits and novel clinical trials for innate immune system-related diseases.

## Data Availability Statement

The datasets presented in this study can be found in online repositories. The names of the repository/repositories and accession number(s) can be found in the article/**Supplementary Material**.

## Author Contributions

YH, YJ, and MA conceived and designed the experiments. MA performed the experiments. YH contributed reagents/materials/analysis tools. MA, YJ, and HJ analyzed the data. MA wrote the first draft of the manuscript. YJ, MK, TE, HJ, and YH revised and edited the manuscript. All authors contributed to the article and approved the submitted version.

## Funding

This research was supported by the Basic Science Research Program through the National Research Foundation of Korea (NRF) funded by the Ministry of Science, ICT and Future Planning (Grant No. 2018R1A2A2A05023367) and by the Korea Institute of Planning and Evaluation for Technology in Food, Agriculture, Forestry and Fisheries (IPET) through the Export Promotion Technology Development Program (Grant no. 617077‐5), funded by the Ministry of Agriculture, Food, and Rural Affairs (MAFRA).

## Conflict of Interest

The authors declare that the research was conducted in the absence of any commercial or financial relationships that could be construed as a potential conflict of interest.

## Publisher’s Note

All claims expressed in this article are solely those of the authors and do not necessarily represent those of their affiliated organizations, or those of the publisher, the editors and the reviewers. Any product that may be evaluated in this article, or claim that may be made by its manufacturer, is not guaranteed or endorsed by the publisher.

## References

[1] Coope G. Several Million Years of Stability Among Insect Species Because of, or in Spite of, Ice Age Climatic Instability? Philos Trans R Soc Lond Ser B Biol Sci (2004) 359(1442):209–14. doi: 10.1098/rstb.2003.1393 PMC1693312 15101577

[2] Ferrandon D Imler J-L Hoffmann JA. Sensing Infection in Drosophila: Toll and Beyond. Semin Immunol (2004) 16(1):43–53. Elsevier. doi: 10.1016/j.smim.2003.10.008 14751763

[3] Grimaldi D Engel MS Engel MS Engel MS. Evolution of the Insects. New York: Cambridge University Press (2005).

[4] Janeway CA Jr Medzhitov R. Innate Immune Recognition. Annu Rev Immunol (2002) 20(1):197–216. doi: 10.1146/annurev.immunol.20.083001.084359 11861602

[5] Medzhitov R Janeway CA. Decoding the Patterns of Self and Nonself by the Innate Immune System. Science (2002) 296(5566):298–300. doi: 10.1126/science.1068883 11951031

[6] Hoffmann JA. The Immune Response of Drosophila. Nature (2003) 426(6962):33–8. doi: 10.1038/nature02021 14603309

[7] Miller JS Nguyen T Stanley-Samuelson DW. Eicosanoids Mediate Insect Nodulation Responses to Bacterial Infections. Proc Natl Acad Sci (1994) 91(26):12418–22. doi: 10.1073/pnas.91.26.12418 PMC45449 7809052

[8] Pech LL Strand MR. Granular Cells Are Required for Encapsulation of Foreign Targets by Insect Haemocytes. J Cell Sci (1996) 109(8):2053–60. doi: 10.1242/jcs.109.8.2053 8856501

[9] Leonard C Ratcliffe NA Rowley AF. The Role of Prophenoloxidase Activation in Non-Self Recognition and Phagocytosis by Insect Blood Cells. J Insect Physiol (1985) 31(10):789–99. doi: 10.1016/0022-1910(85)90072-1

[10] Tauszig S Jouanguy E Hoffmann JA Imler J-L. Toll-Related Receptors and the Control of Antimicrobial Peptide Expression in Drosophila. Proc Natl Acad Sci (2000) 97(19):10520–5. doi: 10.1073/pnas.180130797 PMC27057 10973475

[11] Belvin MP Anderson KV. A Conserved Signaling Pathway: The Drosophila Toll-Dorsal Pathway. Annu Rev Cell Dev Biol (1996) 12(1):393–416. doi: 10.1146/annurev.cellbio.12.1.393 8970732

[12] Weber AN Tauszig-Delamasure S Hoffmann JA Lelièvre E Gascan H Ray KP. Binding of the Drosophila Cytokine Spätzle to Toll Is Direct and Establishes Signaling. Nat Immunol (2003) 4(8):794–800. doi: 10.1038/ni955 12872120

[13] Shia AK Glittenberg M Thompson G Weber AN Reichhart J-M Ligoxygakis P. Toll-Dependent Antimicrobial Responses in Drosophila Larval Fat Body Require Spätzle Secreted by Haemocytes. J Cell Sci (2009) 122(24):4505–15. doi: 10.1242/jcs.049155 PMC2787462 19934223

[14] Ming M Obata F Kuranaga E Miura M. Persephone/Spätzle Pathogen Sensors Mediate the Activation of Toll Receptor Signaling in Response to Endogenous Danger Signals in Apoptosis-Deficient Drosophila. J Biol Chem (2014) 289(11):7558–68. doi: 10.1074/jbc.M113.543884 PMC3953269 24492611

[15] Richman AM Dimopoulos G Seeley D Kafatos FC. Plasmodium Activates the Innate Immune Response of Anopheles Gambiae Mosquitoes. EMBO J (1997) 16(20):6114–9. doi: 10.1093/emboj/16.20.6114 PMC1326295 9321391

[16] Christophides GK Zdobnov E Barillas-Mury C Birney E Blandin S Blass C. Immunity-Related Genes and Gene Families in Anopheles Gambiae. Science (2002) 298(5591):159–65. doi: 10.1126/science.1077136 12364793

[17] Cui C Wang Y Liu J Zhao J Sun P Wang S. A Fungal Pathogen Deploys a Small Silencing RNA That Attenuates Mosquito Immunity and Facilitates Infection. Nat Commun (2019) 10(1):1–10. doi: 10.1038/s41467-019-12323-1 31541102 PMC6754459

[18] An C Jiang H Kanost MR. Proteolytic Activation and Function of the Cytokine Spätzle in the Innate Immune Response of a Lepidopteran Insect, Manduca Sexta. FEBS J (2010) 277(1):148–62. doi: 10.1111/j.1742-4658.2009.07465.x PMC2805777 19968713

[19] Yu B Sang Q Pan G Li C Zhou Z. A Toll-Spätzle Pathway in the Immune Response of Bombyx Mori. Insects (2020) 11(9):586. doi: 10.3390/insects11090586 PMC7564906 32882853

[20] Huerlimann R Wade NM Gordon L Montenegro JD Goodall J McWilliam S. *De Novo* Assembly, Characterization, Functional Annotation and Expression Patterns of the Black Tiger Shrimp (Penaeus Monodon) Transcriptome. Sci Rep (2018) 8(1):1–14. doi: 10.1038/s41598-018-31148-4 30202061 PMC6131155

[21] Roh K-B Kim C-H Lee H Kwon H-M Park J-W Ryu J-H. Proteolytic Cascade for the Activation of the Insect Toll Pathway Induced by the Fungal Cell Wall Component. J Biol Chem (2009) 284(29):19474–81. doi: 10.1074/jbc.M109.007419 PMC2740573 19473968

[22] Park JB Choi WH Kim SH Jin HJ Han YS Kim NJ. Developmental Characteristics of Tenebrio Molitor Larvae (Coleoptera: Tenebrionidae) in Different Instars. Int J Ind Entomol (2014) 28(1):5–9. doi: 10.7852/ijie.2014.28.1.5

[23] Gilbert LI. Insect Molecular Biology and Biochemistry. London: Academic Press (2012).

[24] Ali Mohammadie Kojour M Han YS Jo YH. An Overview of Insect Innate Immunity. Entomol Res (2020) 50(6):282–91. doi: 10.1111/1748-5967.12437

[25] Keshavarz M Jo YH Edosa TT Bae YM Han YS. TmPGRP-SA Regulates Antimicrobial Response to Bacteria and Fungi in the Fat Body and Gut of Tenebrio Molitor. Int J Mol Sci (2020) 21(6):2113. doi: 10.3390/ijms21062113 PMC7139795 32204438

[26] Keshavarz M Jo YH Edosa TT Han YS. Tenebrio Molitor PGRP-LE Plays a Critical Role in Gut Antimicrobial Peptide Production in Response to Escherichia Coli. Front Physiol (2020) 11:320. doi: 10.3389/fphys.2020.00320 32372972 PMC7179671

[27] Hoffmann JA. Innate Immunity of Insects. Curr Opin Immunol (1995) 7(1):4–10. doi: 10.1016/0952-7915(95)80022-0 7772280

[28] Choe KM Werner T Stoven S Hultmark D Anderson KV. Requirement for a Peptidoglycan Recognition Protein (PGRP) in Relish Activation and Antibacterial Immune Responses in Drosophila. Science (2002) 296(5566):359–62. doi: 10.1126/science.1070216 11872802

[29] Gottar M Gobert V Michel T Belvin M Duyk G Hoffmann JA. The Drosophila Immune Response Against Gram-Negative Bacteria Is Mediated by a Peptidoglycan Recognition Protein. Nature (2002) 416(6881):640–4. doi: 10.1038/nature734 11912488

[30] Rämet M Manfruelli P Pearson A Mathey-Prevot B Ezekowitz RAB. Functional Genomic Analysis of Phagocytosis and Identification of a Drosophila Receptor for E. Coli. Nature (2002) 416(6881):644–8. doi: 10.1038/nature735 11912489

[31] Gobert V Gottar M Matskevich AA Rutschmann S Royet J Belvin M. Dual Activation of the Drosophila Toll Pathway by Two Pattern Recognition Receptors. Science (2003) 302(5653):2126–30. doi: 10.1126/science.1085432 14684822

[32] Yang YT Lee MR Lee SJ Kim S Nai YS Kim JS. Tenebrio Molitor Gram-Negative-Binding Protein 3 (TmGNBP3) Is Essential for Inducing Downstream Antifungal Tenecin 1 Gene Expression Against Infection With Beauveria Bassiana JEF-007. Insect Sci (2018) 25(6):969–77. doi: 10.1111/1744-7917.12482 28544681

[33] Vigneron A Jehan C Rigaud T Moret Y. Immune Defenses of a Beneficial Pest: The Mealworm Beetle, Tenebrio Molitor. Front Physiol (2019) 10:138. doi: 10.3389/fphys.2019.00138 30914960 PMC6422893

[34] Patnaik BB Patnaik HH Seo GW Jo YH Lee YS Lee BL. Gene Structure, cDNA Characterization and RNAi-Based Functional Analysis of a Myeloid Differentiation Factor 88 Homolog in Tenebrio Molitor Larvae Exposed to Staphylococcus Aureus Infection. Dev Comp Immunol (2014) 46(2):208–21. doi: 10.1016/j.dci.2014.04.009 24755285

[35] Jo YH Kim YJ Park KB Seong JH Kim SG Park S. TmCactin Plays an Important Role in Gram-Negative and -Positive Bacterial Infection by Regulating Expression of 7 AMP Genes in Tenebrio Molitor. Sci Rep (2017) 7:46459. doi: 10.1038/srep46459 28418029 PMC5394457

[36] Keshavarz M Jo YH Park KB Ko HJ Edosa TT Lee YS. TmDorX2 Positively Regulates Antimicrobial Peptides in Tenebrio Molitor Gut, Fat Body, and Hemocytes in Response to Bacterial and Fungal Infection. Sci Rep (2019) 9(1):16878. doi: 10.1038/s41598-019-53497-4 31728023 PMC6856108

[37] Keshavarz M Jo YH Patnaik BB Park KB Ko HJ Kim CE. TmRelish is Required for Regulating the Antimicrobial Responses to Escherichia Coli and Staphylococcus Aureus in Tenebrio Molitor. Sci Rep (2020) 10(1):4258. doi: 10.1038/s41598-020-61157-1 32144366 PMC7060202

[38] Jang HA Park KB Kim BB Ali Mohammadie Kojour M Bae YM Baliarsingh S. *In Silico* Identification and Expression Analyses of Defensin Genes in the Mealworm Beetle Tenebrio Molitor. Entomol Res (2020) 50(12):575–85 doi: 10.1111/1748-5967.12468

[39] Jo YH Park S Park KB Noh MY Cho JH Ko HJ. *In Silico* Identification, Characterization and Expression Analysis of Attacin Gene Family in Response to Bacterial and Fungal Pathogens in Tenebrio Molitor. Entomol Res (2018) 48(1):45–54. doi: 10.1111/1748-5967.12287

[40] Keshavarz M Jo YH Park KB Ko HJ Edosa TT Lee YS. Tm DorX2 Positively Regulates Antimicrobial Peptides in Tenebrio Molitor Gut, Fat Body, and Hemocytes in Response to Bacterial and Fungal Infection. Sci Rep (2019) 9(1):1–19. doi: 10.1038/s41598-019-53497-4 31728023 PMC6856108

[41] Ali Mohammadie Kojour M Jang HA Edosa TT Keshavarz M Kim BB Bae YM. Identification, *In Silico* Characterization, and Expression Analysis of Tenebrio Molitor Cecropin-2. Entomol Res (2020) 51(2):74–82. doi: 10.1111/1748-5967.12476

[42] Jang HA Park KB Kim BB Ali Mohammadie Kojour M Bae YM Baliarsingh S. Bacterial But Not Fungal Challenge Up-Regulates the Transcription of Coleoptericin Genes in Tenebrio Molitor. Entomol Res (2020) 50(9):440–9. doi: 10.1111/1748-5967.12465

[43] Keshavarz M Jo YH Patnaik BB Park KB Ko HJ Kim CE. Tm Relish Is Required for Regulating the Antimicrobial Responses to Escherichia Coli and Staphylococcus Aureus in Tenebrio Molitor. Sci Rep (2020) 10(1):1–18. doi: 10.1038/s41598-020-61157-1 32144366 PMC7060202

[44] Edosa TT Jo YH Keshavarz M Bae YM Kim DH Lee YS. TmSpz4 Plays an Important Role in Regulating the Production of Antimicrobial Peptides in Response to Escherichia Coli and Candida Albicans Infections. Int J Mol Sci (2020) 21(5):1878. doi: 10.3390/ijms21051878 PMC7084639 32182940

[45] Edosa TT Jo YH Keshavarz M Bae YM Kim DH Lee YS. TmSpz6 Is Essential for Regulating the Immune Response to Escherichia Coli and Staphylococcus Aureus Infection in Tenebrio Molitor. Insects (2020) 11(2):105–19. doi: 10.3390/insects11020105 PMC7074004 32033290

[46] Larkin MA Blackshields G Brown NP Chenna R McGettigan PA McWilliam H. Clustal W and Clustal X Version 2.0. Bioinformatics (2007) 23(21):2947–8. doi: 10.1093/bioinformatics/btm404 17846036

[47] Kumar S Stecher G Li M Knyaz C Tamura K. MEGA X: Molecular Evolutionary Genetics Analysis Across Computing Platforms. Mol Biol Evol (2018) 35(6):1547–9. doi: 10.1093/molbev/msy096 PMC5967553 29722887

[48] Saitou N Nei M. The Neighbor-Joining Method: A New Method for Reconstructing Phylogenetic Trees. Mol Biol Evol (1987) 4(4):406–25. 10.1093/oxfordjournals.molbev.a040454 3447015

[49] Jang HA Patnaik BB Ali Mohammadie Kojour M Kim BB Bae YM Park KB. TmSpz-Like Plays a Fundamental Role in Response to E. Coli But Not S. Aureus or C. Albican Infection in Tenebrio Molitor *via* Regulation of Antimicrobial Peptide Production. Int J Mol Sci (2021) 22(19):10888. doi: 10.3390/ijms221910888 34639230 PMC8509142

[50] Livak KJ Schmittgen TD. Analysis of Relative Gene Expression Data Using Real-Time Quantitative PCR and the 2(-Delta Delta C(T)) Method. Methods (2001) 25(4):402–8. doi: 10.1006/meth.2001.1262 11846609

[51] Goel MK Khanna P Kishore J. Understanding Survival Analysis: Kaplan-Meier Estimate. Int J Ayurveda Res (2010) 1(4):274. doi: 10.4103/0974-7788.76794 21455458 PMC3059453

[52] Moon HJ Lee SY Kurata S Natori S Lee BL. Purification and Molecular Cloning of cDNA for an Inducible Antibacterial Protein From Larvae of the Coleopteran, Tenebrio Molitor. J Biochem (1994) 116(1):53–8. doi: 10.1093/oxfordjournals.jbchem.a124502 7798186

[53] Park S Jo YH Park KB Ko HJ Kim CE Bae YM. TmToll-7 Plays a Crucial Role in Innate Immune Responses Against Gram-Negative Bacteria by Regulating 5 AMP Genes in Tenebrio Molitor. Front Immunol (2019) 10:310. doi: 10.3389/fimmu.2019.00310 30930888 PMC6424196

[54] Anthoney N Foldi I Hidalgo A. Toll and Toll-Like Receptor Signalling in Development. Development (2018) 145(9):dev156018. doi: 10.1242/dev.156018 29695493

[55] Harnish JM Link N Yamamoto S. Drosophila as a Model for Infectious Diseases. Int J Mol Sci (2021) 22(5):2724. doi: 10.3390/ijms22052724 33800390 PMC7962867

[56] Kim CH Kim SJ Kan H Kwon HM Roh KB Jiang R. A Three-Step Proteolytic Cascade Mediates the Activation of the Peptidoglycan-Induced Toll Pathway in an Insect. J Biol Chem (2008) 283(12):7599–607. doi: 10.1074/jbc.M710216200 18195005

[57] Borst DW Bollenbacher WE O’Connor JD King DS Fristrom JW. Ecdysone Levels During Metamorphosis of Drosophila Melanogaster. Dev Biol (1974) 39(1):308–16. doi: 10.1016/S0012-1606(74)80032-1 4211943

[58] Meister M Richards G. Ecdysone and Insect Immunity: The Maturation of the Inducibility of the Diptericin Gene in Drosophila Larvae. Insect Biochem Mol Biol (1996) 26(2):155–60. doi: 10.1016/0965-1748(95)00076-3 8882658

[59] Verma P Tapadia MG. Immune Response and Anti-Microbial Peptides Expression in Malpighian Tubules of Drosophila Melanogaster Is Under Developmental Regulation. PloS One (2012) 7(7):e40714. doi: 10.1371/journal.pone.0040714 22808242 PMC3395640

[60] Zheng W Rus F Hernandez A Kang P Goldman W Silverman N. Dehydration Triggers Ecdysone-Mediated Recognition-Protein Priming and Elevated Anti-Bacterial Immune Responses in Drosophila Malpighian Tubule Renal Cells. BMC Biol (2018) 16(1):60. doi: 10.1186/s12915-018-0532-5 29855367 PMC5984326

[61] Bulet P Hetru C Dimarcq JL Hoffmann D. Antimicrobial Peptides in Insects; Structure and Function. Dev Comp Immunol (1999) 23(4-5):329–44. doi: 10.1016/s0145-305x(99)00015-4 10426426

[62] Ekengren S Hultmark D. Drosophila Cecropin as an Antifungal Agent. Insect Biochem Mol Biol (1999) 29(11):965–72. doi: 10.1016/s0965-1748(99)00071-5 10560137

[63] Tzou P Ohresser S Ferrandon D Capovilla M Reichhart JM Lemaitre B. Tissue-Specific Inducible Expression of Antimicrobial Peptide Genes in Drosophila Surface Epithelia. Immunity (2000) 13(5):737–48. doi: 10.1016/s1074-7613(00)00072-8 11114385

[64] Imler JL Bulet P. Antimicrobial Peptides in Drosophila: Structures, Activities and Gene Regulation. Chem Immunol Allergy (2005) 86:1–21. doi: 10.1159/000086648 15976485

[65] Lemaitre B Hoffmann J. The Host Defense of Drosophila Melanogaster. Annu Rev Immunol (2007) 25:697–743. doi: 10.1146/annurev.immunol.25.022106.141615 17201680

[66] Jo YH Patnaik BB Hwang J Park KB Ko HJ Kim CE. Regulation of the Expression of Nine Antimicrobial Peptide Genes by TmIMD Confers Resistance Against Gram-Negative Bacteria. Sci Rep (2019) 9(1):10138. doi: 10.1038/s41598-019-46222-8 31300668 PMC6626034

[67] Meng X Khanuja BS Ip YT. Toll Receptor-Mediated Drosophila Immune Response Requires Dif, an NF-kappaB Factor. Genes Dev (1999) 13(7):792–7. doi: 10.1101/gad.13.7.792 PMC316597 10197979

[68] Rutschmann S Jung AC Hetru C Reichhart JM Hoffmann JA Ferrandon D. The Rel Protein DIF Mediates the Antifungal But Not the Antibacterial Host Defense in Drosophila. Immunity (2000) 12(5):569–80. doi: 10.1016/S1074-7613(00)80208-3 10843389

[69] Kaneko T Yano T Aggarwal K Lim JH Ueda K Oshima Y. PGRP-LC and PGRP-LE Have Essential Yet Distinct Functions in the Drosophila Immune Response to Monomeric DAP-Type Peptidoglycan. Nat Immunol (2006) 7(7):715–23. doi: 10.1038/ni1356 16767093

[70] Brey PT Lee WJ Yamakawa M Koizumi Y Perrot S François M. Role of the Integument in Insect Immunity: Epicuticular Abrasion and Induction of Cecropin Synthesis in Cuticular Epithelial Cells. Proc Natl Acad Sci USA (1993) 90(13):6275–9. doi: 10.1073/pnas.90.13.6275 PMC46911 8327509

[71] Huttner KM Bevins CL. Antimicrobial Peptides as Mediators of Epithelial Host Defense. Pediatr Res (1999) 45(6):785–94. doi: 10.1203/00006450-199906000-00001 10367766

[72] McGavin GC. Essential Entomology: An Order-by-Order Introduction. New York: Oxford University Press (2001).

[73] McGettigan J McLennan RKJ Broderick KE Kean L Allan AK Cabrero P. Insect Renal Tubules Constitute a Cell-Autonomous Immune System That Protects the Organism Against Bacterial Infection. Insect Biochem Mol Biol (2005) 35(7):741–54. doi: 10.1016/j.ibmb.2005.02.017 15894191

[74] Broderick NA. Friend, Foe or Food? Recognition and the Role of Antimicrobial Peptides in Gut Immunity and Drosophila–Microbe Interactions. Philos Trans R Soc B: Biol Sci (2016) 371(1695):20150295. doi: 10.1098/rstb.2015.0295 PMC4874392 27160597

