# Supplementary material for: Critical Roles of Spätzle5 in Antimicrobial Peptide Production Against Escherichia coli in Tenebrio molitor Malpighian Tubules
Source: Front Immunol. 2021 Dec 16;12:760475. doi: 10.3389/fimmu.2021.760475 (PMC8717915; doi:10.3389/fimmu.2021.760475)
Supplement: Supplementary file 1 [file DataSheet_1.docx]

**Supplementary Table 1. A comparative summary of *TmSpz5*, *TmSpz4*, and *TmSpz6* antimicrobial activity against *E. coli***

| Gene name | *Tm*Spz5 | | | | *TmSpz4* | | | *TmSpz6* | | |
| --- | --- | --- | --- | --- | --- | --- | --- | --- | --- | --- |
| **Tissues** | **Hemocytes** | **Fat bodies** | **Gut** | **MTs** | **Hemocytes** | **Fat bodies** | **Gut** | **Hemocytes** | **Fat bodies** | **Gut** |
| **Activated AMP genes** | *TmTene1*  *TmDef*  *TmAtt2* | *TmTene4*  *TmDef*  *TmTLP1* | *TmAtt1a TmAtt1b* | *TmTene1 TmTene2 TmTene3* *TmTene4 TmCec2 TmColeA TmColeB TmAtt1a TmAtt1b TmAtt2 TmTLP1 TmTLP2* | *TmTen-2* | *TmTen-2 TmTen-3*  *TmTen-4*  *TmAtt-1a*  *TmDef-like*  *TmTLP-1*  *TmCec-2* | *TmTen-4 TmAtt-1a*  *TmCec-2* | *TmTen-2* | *TmTen-3*  *TmDef-like*  *TmTLP-1*  *TmTLP-2*  *TmCec-2* | *TmTen-1*  *TmTen-2*  *TmTen-4*  *TmCol-A*  *TmTLP-2*  *TmDef-like*  *TmCec-2* |
| **References** | Current manuscript | | | | (Edosa et al., 2020a) | | | (Edosa et al., 2020b) | | |


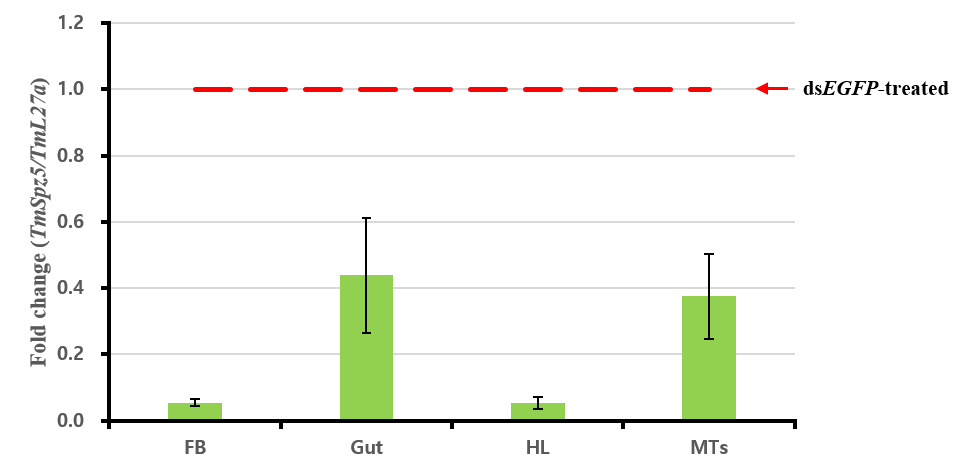


**Supplementary figure 1. Tissue-dependent knockdown efficiency of *TmSpz5*.** The silencing efficiency of ds*TmSpz5* was measured by qRT-PCR at 4 days post-injection. PBS-injected larvae showed no statistically significant differences in survival in both the ds*TmSpz5* and ds*EGFP* groups. The data are reported as averages of three biologically independent replicates.
